# Efficacy and safety of polidocanol in the treatment of varicose veins of lower extremities: A protocol for systematic review and meta-analysis

**DOI:** 10.1097/MD.0000000000024500

**Published:** 2021-02-26

**Authors:** Nan Li, Junhai Li, Mei Huang, Xiujun Zhang

**Affiliations:** Department of Vascular Surgery, Tianjin Hospital, Tianjin, Tianjin, China.

**Keywords:** meta-analysis, polidocanol, RCTs, varicose vein of lower limb

## Abstract

**Background:**

The varicose veins of the lower extremities showed earthworm-like dilatation and venous protrusion of the lower extremities. Polidocanol foam sclerotherapy, as a minimally invasive treatment with rapid recovery, less trauma and not easy to relapse, has achieved good results in clinical, but it is lack of evidence-based medicine. The purpose of this study is to evaluate the efficacy and safety of polidocanol in the treatment of varicose veins of the lower extremities by meta-analysis.

**Method:**

Chinese National Knowledge Infrastructure, Wanfang Database, Chinese Scientifific Journals Database, China Biology Medicine disc, PubMed, EMBASE database, Web of Science, and Cochrane Library will be used as search sources to conduct for randomized controlled trials of polidocanol in the treatment of varicose veins of lower extremities. The search time is set from the establishment of the database in December 2020 in this study. Two researchers independently extract, delete files, extract data and evaluate the quality. Revman software version 5.3 will be used for statistical analysis of data.

**Result:**

In this study, the efficacy and safety of polidocanol in the treatment of varicose veins of the lower extremities will be evaluated in terms of total effective rate, incidence of complications and recurrence rate.

**Conclusion:**

This study will provide reliable evidence-based evidence for the clinical application of polidocanol in the treatment of varicose veins of lower extremities.

**Ethics and dissemination:**

Private information from individuals will not be published. This systematic review also does not involve endangering participant rights. Ethical approval will not be required. The results may be published in a peer-reviewed journal or disseminated at relevant conferences.

**OSF Registration number:**

DOI 10.17605/OSF.IO/AUR4X.

## 1 Introduction

Varicose veins of lower extremities are a common vascular surgical disease. It is usually characterized by the heaviness, pulsation and pain of the lower extremities, which can be observed. Lower limb swelling, venous dilatation, late pigmentation, fatty skin sclerosis and ulcers and other symptoms.^[1]^ It affects more than 20% of the population in developed countries.^[2]^ Its treatment brings serious economic pressure. Due to the lack of understanding of this disease, Chinese patients only receive treatment when they develop into severe venous disease, while Europe and the United States tend to be hospitalized early to prevent serious complications.^[3]^ Varicose veins of lower extremities are mainly caused by valvular insufficiency, weak vein wall and increased pressure of superficial vein.^[4]^ Muscle pump plays a certain role in it. When muscle pump failure is caused by sedentary, neuromuscular degenerative diseases and obesity, it will aggravate venous stagnation and increase venous pressure.^[5]^

The treatment of varicose veins of the lower extremities includes conservative treatment, open surgery and minimally invasive treatment.^[6]^ Conservative treatment includes taking blood-activating drugs, air pressure therapy, wearing medical elastic socks, raising the affected limbs, changing bad living habits, and so on. These treatments can temporarily relieve the symptoms of patients, but cannot solve the fundamental problem.^[7]^ Open surgery includes high ligation and venous exfoliation, which are mostly used for the treatment of trunk veins such as great saphenous vein. However, this treatment will make patients feel huge pain and residual surgical scars, as well as higher treatment costs and longer hospital stay. Minimally invasive surgery includes intracaval radiofrequency ablation, laser ablation and sclerotherapy. In the late 1980 s, the introduction of ultrasound technology brought sclerotherapy into people's field of vision. Because of its accuracy and safety, it has been popularized.^[8]^ Singh et al. show that foam sclerotherapy has less side effects and good contact with the blood vessel wall, so it is the first choice for treatment.^[9]^ Oliveira et al. show that foam sclerotherapy reduces the use of anesthesia and shortens the time of hospitalization and bed rest after treatment.^[10]^

At present, there are a number of randomized controlled studies.^[11–15]^ The results show that polidocanol in the treatment of varicose veins of the lower extremities can significantly relieve symptoms and improve the appearance of legs, eliminate venous reflux and reduce vein diameter, and the operation method is simple and quick, the postoperative recovery is fast, and the adverse events are mild and short-lived. However, there are differences in the research scheme and curative effect of each clinical trial, which leads to uneven results. Therefore, this study plans to systematically evaluate the efficacy and safety of polidocanol in the treatment of varicose veins of the lower extremities, and to provide reliable evidence-based basis for the clinical application of polidocanol in the treatment of varicose veins of lower extremities.

## 2 Methods

### 2.1 Protocol register

This study protocol of systematic review and meta-analysis has been drafted under the guidance of the preferred reporting items for systematic reviews and meta-analyses protocols (PRISMA-P). And, it has been registered on open science framework (OSF) (Registration number: DOI 10.17605/OSF.IO/AUR4X).

### 2.2 Ethics

Since all the data used in this study have been published, there is no need to collect personal information, so the approval of the Ethics Committee is not required. In addition, all data will be anonymously analyzed during the review process.

### 2.3 2.3Eligibility criteria

#### 2.3.1 2.3.1Types of studies

Randomized controlled trials of polidocanol in the treatment of varicose veins of the lower extremities will be included. The literature language is limited to Chinese and English. There is no special requirement that it is single-blind or double-blind.

#### 2.3.2 Research objects

Patients are diagnosed with varicose veins of the lower extremities. The nationality, race, sex and severity of the patients included in the study are not limited, and the incidence of unilateral or bilateral lower extremities will not be limited.

#### 2.3.3 Interventions

The intervention measures of the treatment group are polidocanol or polidocanol combined treatment, and the control group is other treatment methods, the specific treatment will not be limited.

#### 2.3.4 Outcome indicators

Main outcome: Total effective rate: (markedly effective number + effective number) / total (markedly effective CEAP^[16]^ grade rises 1≥grade, clinical symptoms are significantly improved; effective CEAP grade is unchanged. Clinical symptoms were significantly improved.)

Secondary outcome:

(1) incidence of operative complications (subcutaneous congestion, sensory numbness, subcutaneous fat liquefaction infection, skin burn, phlebitis, etc),
(2) intraoperative bleeding volume;
(3) recurrence rate.

### 2.4 Exclusion criteria

(1) Republished literature.
(2) There is no control group in the original literature.
(3) The outcome index does not meet the requirements.
(4) The data of the article are incomplete, so contact the literature which is still not available to the author.

### 2.5 Search strategy

Chinese databases (CNKI, Wanfang, Chinese Scientifific Journals Database, China Biomedical Database) and English databases (PubMed, EMBASE, Web of science, Cochrane Library) will be searched by computer. The search time is set from the establishment of the database to December 2020 in this study, and all the domestic and foreign literature on the treatment of varicose veins of lower extremities will be collected. Chinese search words are “polidocanol,” “lauromacrogol,” “varicose veins of lower extremities” and so on. English search words are “polidocanol”, “lauromacrogol,” “varicose vein” and so on. Take PubMed as an example, the retrieval strategy is shown in Table 1.

**Table 1:** Retrieval strategy of PubMed.

Number	Search terms
1	polidocanol[MeSH]
2	polidocanol[Title/Abstract]
3	lauromacrogol[Title/Abstract]
4	laureth[Title/Abstract]
5	1 OR 2 OR 3 OR 4
6	varix[MeSH]
7	varix[Title/Abstract]
8	Varicose vein [Title/Abstract]
9	6 OR 7 OR8
10	5AND 9

### 2.6 Data screening and extraction

**Figure 1:**
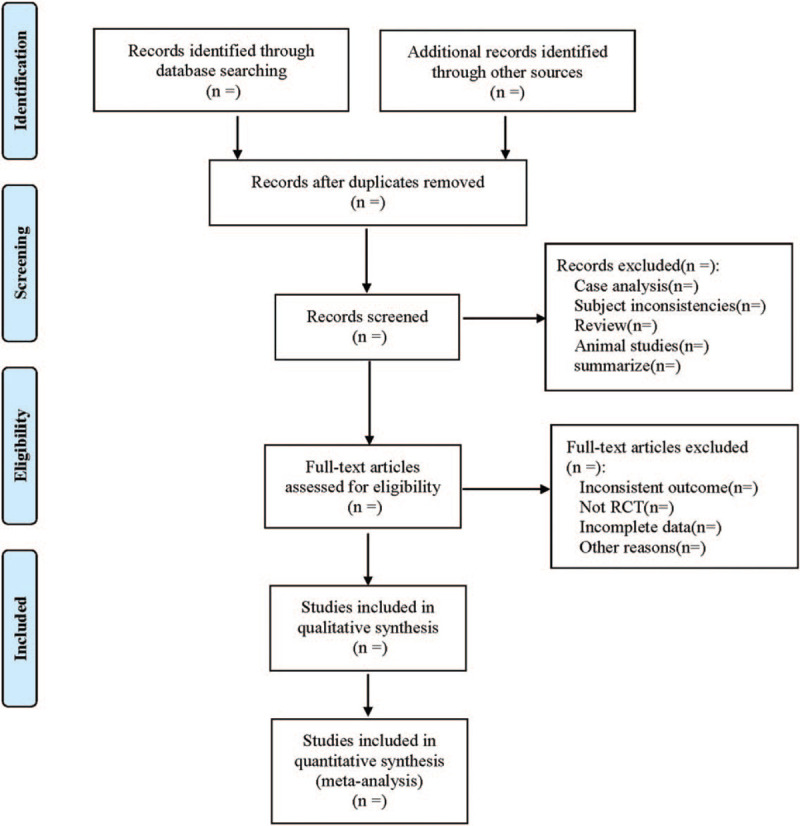
The process of literature screening.

All the documents are included in EndnoteX9 software. After excluding duplicate documents, the 2-person independent extraction method will be used. According to the above-mentioned inclusion and exclusion criteria, documents that are inconsistent with the research theme will be excluded after reading the title and abstract of the document and carefully reading the full text. Cross-check, and resolve the differences through consensus after discussion. The data extracted include: the name of the first author, the year of publication, the number of cases in each group, intervention and control measures, outcome indicators, and so on. The screening process is shown in Figure 1.

### 2.7 Literature quality evaluation

The Cochrane Collaboration network tool will be used to evaluate the quality of the included research. The 2 researchers independently evaluate the literature according to 6 aspects: selection bias, implementation bias, measurement bias, follow-up bias, reporting bias and other biases. The evaluation results can be divided into 3 situations: low risk, high risk and unclear. Cross-check and agree with the third party in case of inconsistency.

### 2.8 Statistical analysis

#### 2.8.1 Data analysis and processing

Revman software version 5.3 will be used for statistical analysis of data. The *χ*^2^ test will be used for heterogeneity among studies. If *I*^*2*^ ≤ *50%, P* ≥ *.1*, the heterogeneity results are not statistically significant, and meta-analysis uses a fixed effect model; if *I2* *>* *50%, P* *<* *.1*, indicating that there is heterogeneity, then look for the causes of heterogeneity, and if possible, conduct a subgroup analysis of heterogeneity factors. If there is only statistical heterogeneity among the studies, but no clinical heterogeneity or no clinical significance, the random effect model is used. The risk ratio statistical analysis will be used for the classified data, and mean difference will be used for the continuous variables, and the 95% confidence interval will be calculated for all the analyses. If the number of studies included is more than 10, a funnel chart is drawn and publication bias is analyzed.

#### 2.8.2 Dealing with missing data

If there are missing data in the article, contact the author by phone or email and complete the data before analysis. If the author cannot be contacted, or if the author has lost the relevant data, no meta-analysis will be performed.

#### 2.8.3 Subgroup analysis

Subgroup analysis will be performed according to CEAP stages of C2, C3, C4, C5 and C6; subgroup analysis will be performed according to the number of varicose limbs in unilateral group and bilateral group; subgroup analysis will be carried out according to the course of treatment.

#### 2.8.4 Sensitivity analysis

Select another research index and change different analysis models to test the stability of the research results.

#### 2.8.5 Assessment of reporting biases

The funnel chart is used to detect the publication offset of each of the above indicators. Publication bias was detected according to whether both sides of the funnel chart are symmetrical.

#### 2.8.6 Evidence quality evaluation

The GRADE scale is used to evaluate the quality of evidence. It includes 5 aspects: bias risk, consistency, directness, accuracy and publication bias. The quality of the evidence will be classified as high, medium, low and very low.

## 3 Discussion

The symptoms of varicose veins in the lower extremities range from mild symptoms such as fatigue, heaviness, itching, leg restlessness, to severe diseases such as edema, skin fat sclerosis and ulcers, which seriously affect people's quality of life. Some studies have shown that varicose veins have a substantial effect on the function and psychology of patients.^[17]^ More than 1/3 people said their work was affected, and more than half said their standing or sitting activities was affected. In addition, physical function and psychological effects of people with higher-than-average symptoms were more obvious. The anatomical and pathophysiological causes of clinical lesions are fluidity venous hypertension caused by venous valve reflux, venous flow obstruction or both.^[18]^ Pathophysiological characteristics of cell level are hypoxia, imbalance of apoptosis and changes of extracellular matrix.^[19]^ Not only is the quality of life affected, the presence of varicose veins also increases the risk of superficial venous thrombosis and venous thromboembolism disease.^[20]^

Polidocanol sclerotherapy is injected into the varicose vein to damage the vein endothelium, and then fall off to form thrombus, resulting in vein wall hypoxia secondary inflammation, granulation tissue followed by fibrosis growth, varicose vein closure, and finally the formation of fiber cord, to achieve the purpose of treatment.^[21]^ And polidocanol belongs to ether compounds, which has anesthetic effect to a certain extent and can reduce the pain response of the body.^[22]^ Although sclerotherapy was originally liquid sclerotherapy. It gradually evolved into foam sclerotherapy. Because the liquid mixes with the blood quickly, the preparation is diluted, inactivated and passivated. Compared with liquid preparation, foam preparation can avoid mixing with intravascular blood and dislocation with target vessels.^[23]^ Foam hardener refers to the gas-liquid equilibrium preparation made by fully mixing liquid polidocanol with a certain proportion of air or carbon dioxide.^[24]^ Wan Xia et al reported that the therapeutic effect of polycinnamyl alcohol foam sclerotherapy was not inferior to that of intracavitary electrocoagulation and routine exfoliation, and reduced the recurrence rate.^[25–26]^ However, there are also some complications in the course of treatment, such as pigmentation, pain at the injection site, superficial thrombophlebitis, local induration of injection, transient dry cough and so on.^[27]^ The European Association for foam Sclerosing Therapy recommends that the safe dosage of foaming sclerosing agent is 6 to 8 mL, and the conventional dosage is 40 mL within.^[28]^ Serious adverse reactions can be avoided by controlling the dosage.

We hope to conduct a meta-analysis of the existing randomized controlled trials of polidocanol in the treatment of varicose veins of lower extremities, objectively evaluate its safety and efficacy, and provide reliable evidence-based basis for clinical application. However, due to the lack of a large number of books and high-quality randomized controls, the follow-up time is generally short. The therapeutic effect of polidocanol on Varicose veins of lower extremities still needs to further expand the sample size, improve the research quality, prolong the follow-up time and so on. Due to the limitation of language ability, we only search English and Chinese literature and may ignore studies or reports in other languages.

## Author contributions

**Data collection**: Nan Li and Junhai Li.

**Funding support**: Xiujun Zhang.

**Resources**: Junhai Li and Mei Huang.

**Software operating**: Mei Huang and Xiujun Zhang.

**Supervision**: Mei Huang.

**Writing – original draft**: Nan Li and Junhai Li.

**Writing – review & editing**: Nan Li and Xiujun Zhang.
